# Genome Sequence of *Halanaerobium saccharolyticum* subsp. *saccharolyticum* Strain DSM 6643^T^, a Halophilic Hydrogen-Producing Bacterium

**DOI:** 10.1128/genomeA.00187-13

**Published:** 2013-05-02

**Authors:** Anniina Kivistö, Antti Larjo, Alessandro Ciranna, Ville Santala, Christophe Roos, Matti Karp

**Affiliations:** Department of Chemistry and Bioengineering, Tampere University of Technology, Tampere, Finlanda;; Department of Signal Processing, Tampere University of Technology, Tampere, Finlandb;; Department of Information and Computer Science, Aalto University, Helsinki, Finlandc

## Abstract

*Halanaerobium saccharolyticum* is a halophilic anaerobic fermentative bacterium capable of producing hydrogen, a potential future energy carrier molecule. The high-quality draft genome of *H. saccharolyticum* subsp. *saccharolyticum* strain DSM 6643^T^ consists of 24 contigs for 2,873,865 bp with a G+C content of 32.3%.

## GENOME ANNOUNCEMENT

*Halanaerobium saccharolyticum* is a halophilic anaerobic fermentative bacterium belonging to the order *Halanaerobiales* and the family *Halanaerobiaceae* (1, 2). *H. saccharolyticum* is an interesting bacterium due to its efficient hydrogen (bioenergy molecule) production (3, 4), vitamin B_12_-dependent 1,3-propanediol (building block of polymers)-producing pathway that competes with hydrogen production (4, 5), and ability to utilize unpurified raw glycerol, a by-product of traditional biodiesel industry, as a substrate of fermentation (A. Kivistö, A. Ciranna, V. Santala, and M. Karp, submitted for publication). The genome of *H. saccharolyticum* was sequenced in order to provide insight into the halophilic fermentative metabolic pathways. Furthermore, three bacterial species of the family *Halanaerobiaceae* were sequenced recently (6–8). The genome sequence of *H. saccharolyticum*, together with those of the previously sequenced strains, provides valuable information on the adaptation strategies of this group of halophilic fermentative bacteria.

The genome of *H. saccharolyticum* subsp. *saccharolyticum* strain DSM 6643^T^ was sequenced using Illumina paired-end sequencing and 454 technologies, assembled using MIRA (9), and manually edited with Gap5 (10) to fix sequencing and assembly errors and combine contigs. The RAST server (11; http://rast.nmpdr.org/) was used for annotation, and when needed, manual checking and revision of autoannotation were done according to BLAST analysis (12). The resulting “improved high-quality draft” (13) genome is 2,873,865 bp in size, comprising 24 contigs (>1 kb). The genome was predicted to contain 2,664 coding sequences, of which 72 are for RNAs. The maximum contig length in the assembly is 900,505 bp, and the *N*_50_ is 723,182 bp. The G+C content of the genome is 32.3%.

The glycerol fermentation pathways of *H. saccharolyticum* were reconstructed according to genome sequence analysis. The reconstruction revealed hydrogen, carbon dioxide, acetate, butyrate, butanol, ethanol, lactate, malate, and 1,3-propanediol (a vitamin B_12_-dependent route) as putative fermentation products. Four [FeFe]-hydrogenases, of which two are putative bifurcating hydrogenases requiring both reduced ferredoxin and NADH, were identified. The putative bifurcating hydrogenases are suggested to be involved in high-yield H_2_ production. Furthermore, the genes for a multidrug efflux pump (Acr type), β-lactamase, mercuric reductase, a copper-translocating ATPase, and a cobalt-zinc-cadmium resistance protein suggest that *H. saccharolyticum* is resistant to a wide variety of antibiotics and toxic compounds, including heavy metals.

### Nucleotide sequence accession numbers. 

This whole-genome shotgun project has been deposited at DDBJ/EMBL/GenBank under the accession no. CAUI00000000. The version described in this paper is the first version, CAUI01000000.
